# Eosinophilic ulcer mimicking malignancy of the lower lip: A case report

**DOI:** 10.1002/ccr3.2746

**Published:** 2020-03-03

**Authors:** Ghasem Rahmatpour Rokni, Sidharth Sonthalia, Dipali Rathod, Torello Lotti, Mohamad Goldust

**Affiliations:** ^1^ Mazandaran University of Medical Sciences Sari Iran; ^2^ Skinnocence The Skin Center and Reaearch Centre Gurgaon India; ^3^ Consultant Dermatologist Mumbai India; ^4^ University of Studies Guglielmo Marconi Rome Italy; ^5^ University of Rome Guglielmo Marconi Rome Italy; ^6^ Department of Dermatology University Medical Center Mainz Mainz Germany; ^7^ Department of Dermatology University Hospital Basel Basel Switzerland

**Keywords:** eosinophilic ulcer, lip, malignancy

## Abstract

Eosinophilic ulcer (EU) is a rare self‐limiting chronic benign lesion of the oral mucosa. It is an uncommon and benign disease, which may leading to diagnostic difficulties. Biopsy is recommended to rule out any malignant etiology.

## Graphical abstract

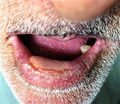

## 1 INTRODUCTION

Squamous cell carcinoma (SCC) represents one of the most common cancers in the head and neck region among the adults. Chronic oral ulcers may at times mimic SCC, and hence, malignancy should be included in the differential diagnosis. Eosinophilic ulcer of the oral mucosa (EUOM) is a scarce, self‐resolving, chronic benign ulcer commonly affecting the oral mucosa. Tongue is the most commonly affected site and is clinically characterized by the presence of a well defined nonhealing ulcer with indurated borders, which may resemble malignancy, traumatic ulceration, and infections like deep fungal infection, tuberculosis, and primary syphilis.^1^, ^2^, ^3^, ^4^, ^5^ The pathogenesis of EU still remains unclear; however, the ulcer is thought to be reactive since trauma has been implicated as the initiating factor.^5^

## 2 CASE PRESENTATION

**Figure 1:**
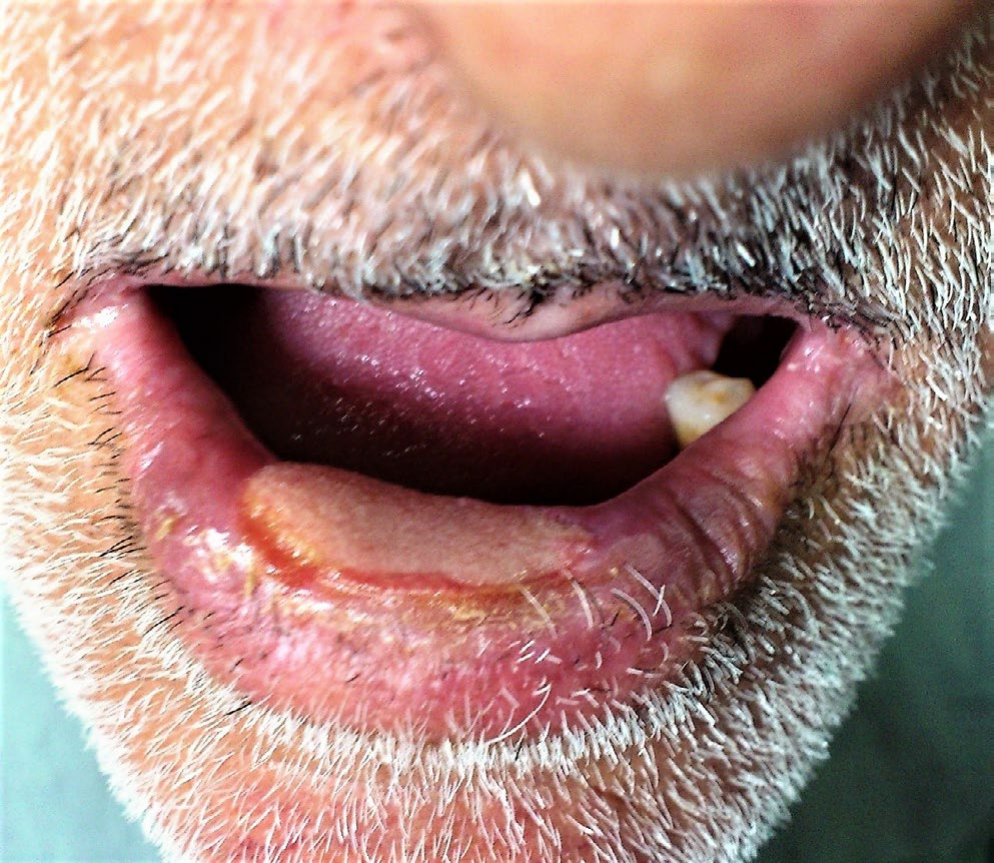
The central portion of the lower lip containing massive indurated painless ulcer covered with slough and yellowish crust at the edges

A 62‐year‐old man, with a large nonhealing ulcer of the lower lip since 1 month, was admitted to our clinic. The ulcer presented initially as an inflammatory papule, which gradually transformed into an ulcer with elevated borders smeared with serous fluid. The patient received outpatient treatment consisting of tablet cephalexin (500 mg QID for 7 days) and tablet acyclovir (400 mg 5 times a day for 4 days); however, the patient's clinical condition failed to improve. He denied any history of physical or chemical injuries. However, for the past 4 years, he was suffering from a psychiatry disorder and his drug history included tablet sertraline 50 mg daily. He was not a smoker and did not use any illicit drugs. His other medical history was insignificant. The extra‐oral examination on admission revealed no abnormality. However, the intra‐oral examination revealed an indurated painless oval ulcer measuring 2 × 1.2 cm in diameter with elevated borders in the central portion of the lower lip with a fibrinous base. The vermilion border adjacent to the ulcer showed desquamation. He had poor oral hygiene with generalized stains, attrition, and carious teeth. The local lymph nodes accessible to physical examination were not enlarged (Figure 1). Routine laboratory evaluations did not show any significant abnormal findings. The enzyme‐linked immuno‐sorbent assay (ELISA) for serum antibodies to HSV‐1 and HSV‐2 and the rapid plasma reagin (RPR) test for syphilis were both negative. Taking the history into account, a clinical differential diagnosis of traumatic and malignant ulcer was made.

**Figure 2:**
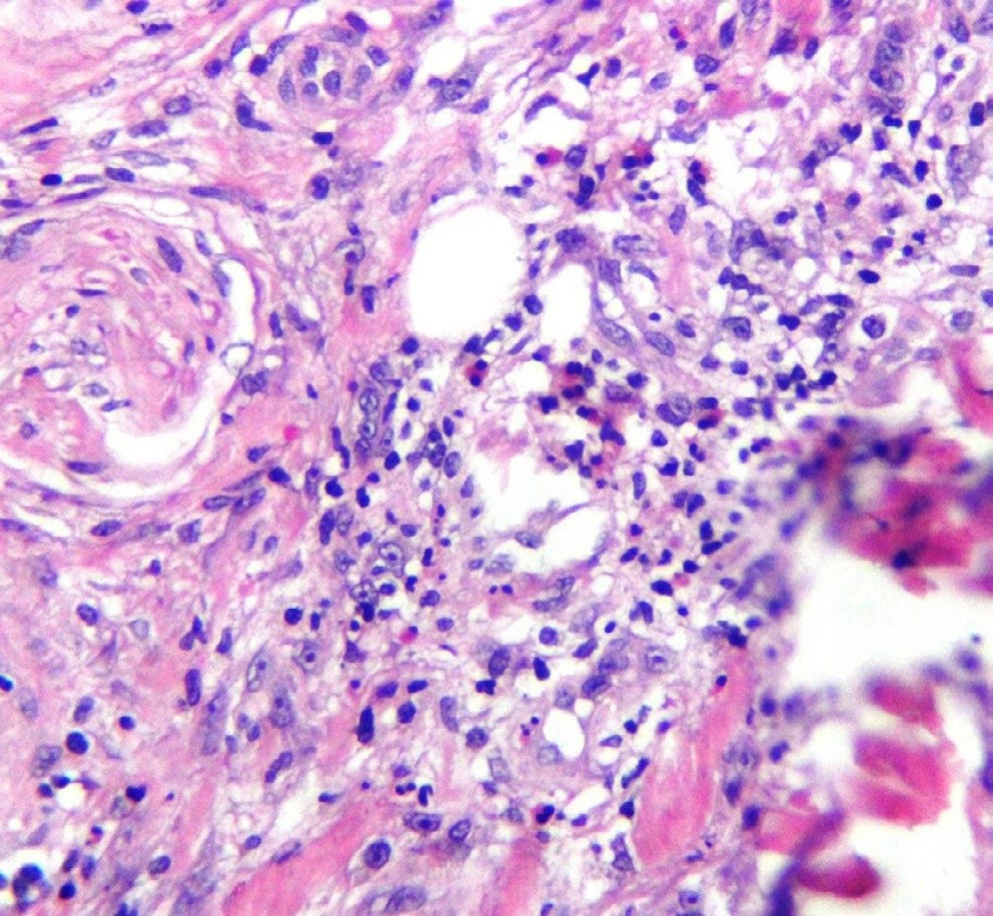
Diffuse infiltration of lymphohistiocytes and eosinophils in the dermis (×400)

**Figure 3:**
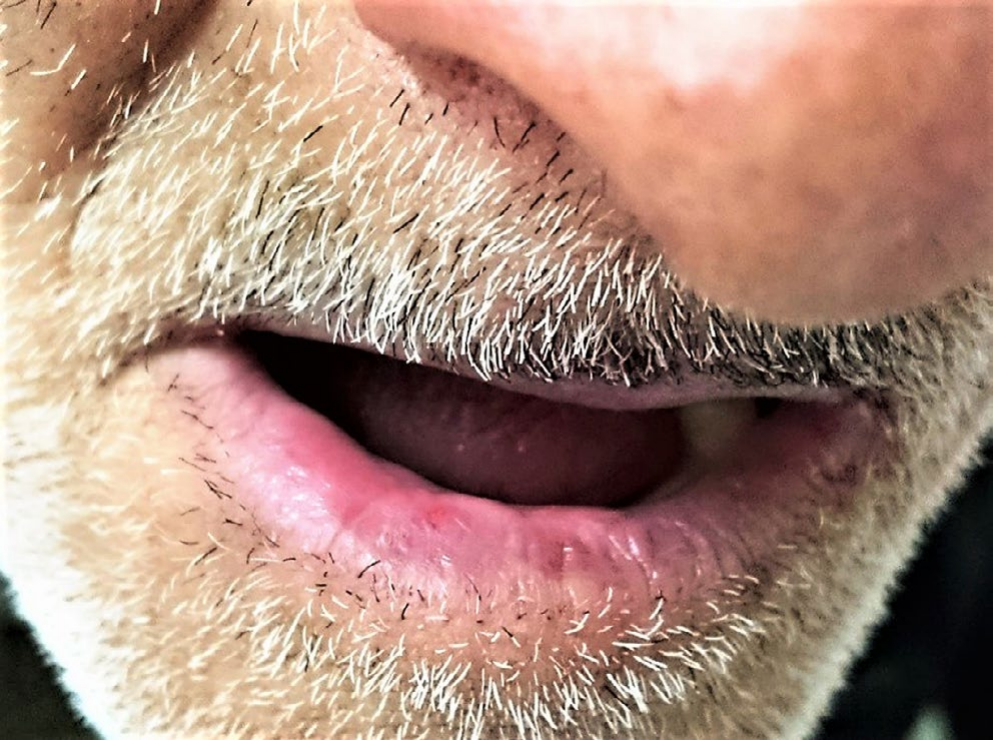
Complete resolution of ulcer after 3 mo without any medical treatment

A deep tissue biopsy was done from the lesion under local anesthesia and sent to two independent pathology laboratories for routine histopathological examination and was stained with hematoxylin and eosin. The histopathology findings revealed ulceration in the epidermis sloughed stratified squamous epithelium with granulation tissue in the dermis composed of fibroblastic and vascular proliferation with a dense, diffuse infiltration of lymphohistiocytes and eosinophils that extended deeply into the submucosal tissue. The above classical features were consistent with EUOM (Figure 2). The ulcer resolved spontaneously after 3 months by only reassuring the patient without any medical treatment (Figure 3).

## 3 DISCUSSION

Eosinophilic ulcer of the oral mucosa (EUOM) is believed to be caused by injury and is a relatively rare, benign mucosal disorder, in contrast to the other types of post‐traumatic ulcerations, which take much longer to heal spontaneously and may persist for over a year.^6^ Self‐inflicted injuries (toothbrush abrasion, sharp‐edged teeth, ill‐fitting dentures, orthodontic appliances) are believed to be the most common EUOM triggers.^7^ Multiple reports state that EUOM is caused by T cell–mediated immune response and may constitute a rare manifestation of drug or allergic reactions in response to currently unknown factors, such as viral particles or toxic products of bacterial cell degradation.^5^, ^6^ Another theory suggests that it represents a CD30+ lymphoproliferative disorder (a type of lymphoma).^7^ The tongue is the most common location of EUOM. Differential diagnosis of oral mucosal ulcers may be very wide and complex; hence, biopsy is recommended in the majority of cases.^8^

Eosinophilic ulcer of the oral mucosa clinically manifests as a rapidly enlarging oral ulcer with indurated margins that closely simulates oral squamous cell carcinoma. Arriving at the diagnosis of ulcerative lesions of the oral mucosa is usually difficult as diverse conditions may share a similar clinical appearance. However, performing a biopsy can easily aid in the diagnosis of EUOM ruling out malignancy and other differential diagnosis.^9^

Apart from malignancy, other clinical differential diagnosis of EUOM that should be considered include affection of oral mucosa by several infectious disorders, for example, granulomatous mycobacterial (oral tuberculosis), primary syphilis (syphilitic chancre), or even necrotizing bacterial infections and fungal (histoplasmosis); granulomatous infections like sarcoidosis, Langerhans’ cell histiocytosis, Wegener's granulomatosis, and discoid lupus erythematosus (DLE).^10^, ^11^

A number of investigations have shown atypical cells seen histopathologically in certain EUOM patients to be T lymphocytes with strong immunoperoxidase reactivity for CD30.^12^, ^13^ T‐cell monoclonality has been found in certain EUOM cases with polymerase chain reaction analysis of the T‐cell receptor (TCR) gamma chain gene; thus, it has been speculated that such cases represent the oral counterpart of the primary cutaneous CD30+ T‐cell lymphoproliferative disorder.^13^, ^14^ However, yet another molecular study of the TCR gamma chain gene in EUOM cases demonstrated its polyclonal, oligoclonal, and least frequently, monoclonal rearrangement.^4^ Rarely recurrence is reported and since monoclonal cases need long‑term follow‐up, these cases should be subjected to immunohistochemical analysis for CD30 marker clonality.^1^

The most interesting aspect regarding both pathology and pathogenesis of EUOM is that this disorder has the tendency for self‐resolution, even in patients with CD30+ cells and TCR gamma chain gene monoclonality. In our patient, the immunoperoxidase staining did reveal CD30‐cells. Histopathology remained gold standard in EUOM diagnosis, and the eosinophils, are a pathological hallmark of EUOM, following their initially deleterious activation, provide a stimulus for self‐resolution of previously damaged/ traumatized tissue. Also, whether self‐resolution of EUOM could be related to the interplay between oral microbial flora and eosinophil remains unclear.^15^ The lesions show a tendency to heal spontaneously, which usually occurs within a month after the onset of symptoms.^6^, ^7^ Treatment with NSAIDs (eg, ibuprofen), local anesthetics (lignocaine, benzocaine, etc), and topical disinfectants usually suffices in majority of cases.^6^ There are contradictory opinions on the usefulness of glucocorticosteroids (GCS) in the treatment of EUOM, as the healing of the lesions may be impaired by the immunosuppressive action of GCS. In cases where secondary bacterial superinfection develops, antibiotic therapy based on sensitivity testing result seems appropriate. If the ulcer fails to heal within 2 weeks of topical treatment, a biopsy is indicated. In many cases, the trauma associated with surgical intervention such as a biopsy, triggers the healing process,^7^ and the lesions rarely require surgical management.^6^ In periods of increased pain, the patient should be advised to switch to liquid or semiliquid diet and avoid hot and sour foods. The prognosis in EUOM is good.^7^ According to the available literature, recurrences seem to be exceptionally rare, although long‐term follow‐up should be recommended if there are pathologic and/ or molecular features of a lymphoproliferation.

## 4 CONCLUSION

Diagnosis and monitoring of this entity is of utmost importance due to its close resemblance to an oral malignancy (eg, squamous cell carcinoma and low‐grade lymphoma) and other misconceived different entities, owing to its presentation as an indurated ulcer in 5th or 6th decade individuals. Our case presented EU on the lower lip, which is a rare presentation and showed complete resolution of the ulcer after 3 months without any medical intervention. The biopsy still remains the best approach for both the diagnostic and therapeutic management; however, the reason for its disappearance after biopsy remains a mystery.

## CONFLICT OF INTEREST

None.

## AUTHOR CONTRIBUTIONS

All authors have read and approved the manuscript, and ensure that this is the case. GRR: involved in patient follow‐up and management and final approval of the version to be published. SS: involved in patient management and writing article, drafted the work, and substantively revised it. DR and MG: involved in patient follow‐up and management drafted the work and substantively revised it. TL: involved in patient management and drafting the manuscript.
